# Sensory experience in obsessive compulsive disorder - sensiocd: Do they think or feel differently?

**DOI:** 10.1192/j.eurpsy.2021.377

**Published:** 2021-08-13

**Authors:** I. Soares Da Costa, V. Covelo, R. Moreira

**Affiliations:** Psychiatry And Mental Health Department, Centro Hospitalar e Universitário de São João, Porto, Portugal

**Keywords:** ocd, sensory experience, obsessive spectrum, obsessive thoughts

## Abstract

**Introduction:**

Obsessive-compulsive disorder (OCD) is associated to a wide range of symptomatic expression and treatment response variability [1]. Sensory perception has been identified as an emerging factor in this process [2]. Sensory vulnerability and atypical sensory experience were identified as risk factors for the development of OCD [3] and a sensory subtype of the disease was proposed in which there is a positive correlation with early onset sensory symptoms, male gender and family background [4]. Adding to the atypical sensory profile, obsessions are frequently experienced as partially perceptual.

**Objectives:**

Our main goals are to characterize the sensory perception in OCD patients; assess the prevalence and intensity of the sensory properties of the obsessive thoughts and explore the how sensory perception, obsessive thoughts and obsessive dimensions/clusters are interrelated.

**Methods:**

Patients with OCD diagnosis, aged 18 to 65 years and no comorbid mental disorder (except depression) will be recruited. The study battery will include participant form with demographical and clinical features, assessment of depressive and anxiety symptoms (HAM-A and HAM-D) evaluation of clinical outcome measures and obsessive dimensions/clusters (Yale-Brown Obsessive-Compulsive Scale (Y-BOQS) and Obsessive Beliefs Questionnaire-44 (OBQ-44)), assessment of sensory perception and sensory properties of obsessive thoughts (Sensory Perception Quotient (SPQ 21) and Sensory Properties of Obsessive Thoughts Questionnaire (SPOQ)).

**Results:**

The results will help us understand the interaction between perceptual and cognitive processes in OCD.

**Conclusions:**

Better definition of OCD psychopathology and the establishment of a sensory subtype may indicate the need of specific therapeutic indications or a different escalation of treatment measures.

**Disclosure:**

No significant relationships.

